# Cohort analysis of high-risk HPV infection in adult women in Dapeng New District, Shenzhen, Guangdong Province, China

**DOI:** 10.3389/fmicb.2025.1539209

**Published:** 2025-03-12

**Authors:** Weifeng Wei, Mi Zhang, Yiyuan Lin, Ziyin Li, Wenbo Luo, Weihua Zhao, Jing Zhuang, Weining Zhao, Zhixue Xu, Gaomin Li, Wenjing Zeng, Liping Huang, Yan Tan, Zhiying Yu, Guanglei Li

**Affiliations:** ^1^Shenzhen Dapeng New District Maternity and Child Health Hospital, Shenzhen, China; ^2^Guangxi University of Chinese Medicine, Nanning, Guangxi, China; ^3^Shenzhen Second People's Hospital, The First Affiliated Hospital of Shenzhen University, Shenzhen, Guangdong, China; ^4^College of Pharmacy, Shenzhen Technology University, Shenzhen, China; ^5^College of Medicine, Shenzhen University, Shenzhen, China; ^6^Dapeng New District Center for Disease Control and Prevention, Shenzhen, Guangdong, China; ^7^Department of Gynecology, Shenzhen Second People's Hospital/The First Affiliated Hospital of Shenzhen University Health Science Center, Shenzhen, China

**Keywords:** Shenzhen Dapeng New District, human papillomavirus (HPV), infection rate, cervical cancer screening, human papillomavirus vaccine vaccination

## Abstract

**Introduction:**

Cervical cancer is the fourth most commonly diagnosed malignancy among women globally, with HPV infection being the primary cause. Large-scale HPV screening is crucial for early detection, as appropriate intervention in HPV-positive individuals can significantly reduce cervical cancer incidence and mortality.

**Methods:**

Data were extracted from the Shenzhen Maternal and Child Health Management Information System, covering 18,667 HPV infection and cervical cancer screening instances among 15,850 women from January 1, 2020, to December 31, 2023. HPV prevalence and corresponding 95% confidence intervals (CIs) were calculated using SPSS (version 27.0).

**Results:**

From January 2020 to December 2023, the overall HPV infection rate was 12.39%, with a notably higher rate of 23.38% in women over 60 years. The hr-HPV infection rate was 12.99%, with HPV types 52, 58, 16, 51, and 68 being the most common. Among women with positive ThinPrep Cytologic Test (TCT) results, the hr-HPV infection rate was 53.37%. In women with positive cervical histopathology, the hr-HPV infection rate reached 95.95%. With increased HPV vaccination doses, the infection rates of HPV16, 18, 52, 58, 51, 31, 33, 45, 6, and 11 decreased among individuals under 30, but increased in those over 45.

**Discussion:**

These findings suggest that enhancing HPV vaccination coverage and cervical cancer screening, especially for women over 45, are effective strategies to reduce hr-HPV infection rates and cervical cancer incidence in this region.

## 1 Introduction

Cervical cancer is the fourth most common cancer worldwide in terms of both incidence and mortality among women, with an estimated 660,000 new cases and 350,000 deaths globally in 2022 (Ferlay et al., 2021). It accounts for over 90% of HPV-related cancers in women (de Martel et al., 2020). Extensive research has been conducted over the years to investigate the etiology and risk factors of cervical cancer, with high-risk human papillomavirus (hr-HPV) infection recognized as the most critical factor (National Health Commission of the People's Republic of China, 2022). In China, current consensus guidelines recommend HPV vaccination for adolescents and adult women as a preventive measure against cervical cancer (Gynecological Oncology Society of Chinese Medical Association, 2021). However, there is limited regional data on the changes in HPV infection rates following large-scale vaccination programs in the country. Therefore, it is necessary to investigate the prevalence of hr-HPV, the distribution characteristics of different HPV subtypes in specific regions, the subtype characteristics of hr-HPV in populations with cervical intraepithelial neoplasia and cervical cancer, and the changes in HPV subtype infection rates following widespread vaccination.

## 2 Materials and methods

### 2.1 Basic information of the study subjects

Data were retrieved from the Shenzhen Maternal and Child Health Management Information System for cervical cancer screening and treatment results of 15,850 women who underwent cervical cancer screening at medical institutions in the Dapeng New District of Shenzhen from 1 January 2020 to 31 December 2023. These women were aged between 20 and 78 years, with a total of 18,667 screening instances. Among them, 1,267 instances used ThinPrep Cytologic Test (TCT) screening; 1,993 instances used HPV nucleic acid typing detection combined with TCT screening; 15,407 instances (82.53%) used HPV nucleic acid typing detection for triage, with positive cases (except HPV16 and 18 positive) undergoing combined TCT screening. Colposcopy triage was performed for those meeting any of the following conditions: (1) HPV16 or 18 positive, (2) persistent hr-HPV infection for ≥2 years, (3) TCT results showing Atypical Squamous Cells of Undetermined Significance (ASC-US) or higher, (4) presence of cervical cancer-related symptoms and signs deemed necessary for colposcopy by the attending physician. The screening process was completed by gynecologists trained in cervical cancer prevention and control knowledge, and colposcopy examinations were performed by gynecologists trained in colposcopy. This study is a retrospective study, and data retrieval was authorized by the Dapeng New District Maternal and Child Health Hospital and ethically approved by the Ethics Committee of Shenzhen Second People's Hospital.

In the HPV screening study, basic demographic information of women was investigated, including age, household registration, and marital status (Table 1).

**Table 1:** Basic demographic information of women undergoing HPV screening in Dapeng New District.

		**Proportion**	**Infection rate (95%CI)**
**Age, year**			
≤25	159	0.91%	23.27% (16.63%−29.91%)
26–30	874	5.02%	12.36% (10.17%−14.54%)
31–35	3,205	18.42%	11.17% (10.08%−12.26%)
36–40	3,553	20.42%	10.67% (9.65%−11.68%)
41–45	3,246	18.66%	11.40% (10.30%−12.49%)
46–50	3,198	18.38%	12.38% (11.24%−13.52%)
51–55	2,135	12.27%	15.97% (14.42%−17.53%)
56–60	953	5.48%	15.53% (13.23%−17.83%)
>60	77	0.44%	23.38% (13.71%−33.05%)
**Marital status**			
Married	16,482	94.72%	12.35% (11.85%−12.86%)
Spinsterhood	674	3.87%	11.42% (9.02%−13.83%)
Unknown	244	1.40%	17.21% (12.44%−21.98%)
**Census register**			
Shenzhen household residence	6,565	37.73%	10.39% (9.65%−11.13%)
Non-Shenzhen household registration	10,805	62.10%	13.60% (12.95%−14.24%)
Unknown	30	0.17%	13.33% (0.42%−26.24%)

### 2.2 Data redundancy analysis

In the raw dataset, 1,469 women (1,550 redundant records) underwent two or more HPV tests. The hr-HPV positivity rate in redundant records was 7.8%. A Kruskal-Wallis test comparing infection rates between redundant and non-redundant data showed no statistically significant differences (*P* > 0.05). Adjusted infection rate differences for all HPV genotypes were less than 1% (Supplementary Table S1). Thus, redundant data did not significantly bias the overall infection rate estimates.

### 2.3 Human papillomavirus testing

Cervical samples for HPV testing were collected using a sterile cervical brush. The brush was gently inserted into the endocervical canal and rotated 5–6 times in a clockwise direction to ensure adequate sample collection, in accordance with established sampling protocols. The collected material was then transferred into a vial containing preservation solution to maintain sample integrity. The vials were appropriately labeled and stored at a controlled temperature until processing. The samples were subsequently prepared for HPV DNA testing using an HPV nucleic acid detection and genotyping kit (HE AS bio Tech, Guangzhou, China), following standardized laboratory procedures. The test results included the identification of 16 human papillomavirus genotypes, comprising 14 high-risk types (HPV 16, 18, 31, 33, 35, 39, 45, 51, 52, 56, 58, 59, 66, and 68) and 2 low-risk types (HPV 6, 11).

### 2.4 HPV vaccination trends and impact on HPV infection rates

During the period from 2020 to 2023, a total of 34,748 doses of HPV vaccine were administered to women in Dapeng New District. Among these, 25,765 doses (74.14%) were administered to women aged 9–35 years (Table 2).

**Table 2:** Age-specific and annual distribution of HPV vaccination doses administered to women in Dapeng New District (2020–2023): focus on coverage in the 9–35-year-old population.

**Year**	**Number**	**Doses for ages 9–35**	**Proportion (%)**
2020	2,206	1,839	83.36
2021	6,002	3,795	63.22
2022	10,510	6,744	64.16
2023	16,030	13,387	83.51
Total	34,748	25,765	74.14

To evaluate the protective effect of large-scale HPV vaccination on specific populations in Dapeng New District, considering that 74.14% of HPV vaccine doses were administered to women aged 9–35 years, we analyzed 17,400 HPV screening tests from 2020 to 2023, dividing the population into three age groups: Group C1 (≤30 years), Group C2 (31–45 years), and Group C3 (>45 years). We calculated the annual trends in infection rates for HPV types 16, 18, 31, 33, 45, 52, 58, 6, and 11 in each group.

### 2.5 Thinprep cytologic test

Cervical specimens for liquid-based cytology were collected and processed to prepare slides for analysis in accordance with standardized protocols (Belinson et al., 2003). The slides were then subjected to a comprehensive cytological review conducted by a trained pathologist. Diagnoses were made according to the grading criteria of the Bethesda System: (1) no intraepithelial or malignant lesions (NILM); (2) squamous epithelial cell abnormality: a, atypical squamous cells (ASCs), including ASC-US and atypical squamous cells that cannot exclude high-grade squamous intraepithelial lesion (ASC-H); b, low-grade squamous intraepithelial lesion (LSIL); c, high-grade squamous intraepithelial lesion (HSIL); and d, squamous cell carcinoma (SCC); (3) glandular epithelial cell abnormality: a, atypical glandular cells (AGCs), including AGC-not otherwise specified (AGC-NOS) and AGC-suspicious for neoplasia (AGC-FN); b, cervical adenocarcinoma *in situ* of the cervical canal (AIS); and c, adenocarcinoma; and (4) other malignant tumors.

### 2.6 Colposcopy and pathological diagnosis

For patients with abnormal HPV and TCT results, colposcopy is performed following the American Society for Colposcopy and Cervical Pathology (ASCCP)guidelines. In necessary cases, cervical biopsy and Endocervical Curettage (ECC) are carried out. Biopsy samples are collected under colposcopy guidance and sent for pathological examination. The pathological specimens are processed by the pathology department and reviewed by two experienced pathologists. In cases where there is a discrepancy between the initial diagnosis and the review, the final judgment is made by the head of the pathology department.

Cervical lesion classification is based on pathological diagnosis, which includes: (1) normal or inflammation; (2) low-grade squamous intraepithelial lesion (LSIL) (Cervical Intraepithelial Neoplasia Grade I, CIN I); (3) high-grade squamous intraepithelial lesion (HSIL), which includes both moderate (Cervical Intraepithelial Neoplasia Grade II, CIN II) and severe (Cervical Intraepithelial Neoplasia Grade III, CIN III) dysplasia; and (4) cervical cancer (CC).

### 2.7 Statistical analysis

Statistical analysis was conducted using SPSS (version 27.0) and Excel (version 2022). HPV positivity for one, two, or more genotypes was defined as single or multiple HPV genotype infections. SPSS 27.0 for Windows (SPSS Inc., IL, USA) was used to calculate the HPV infection rates and corresponding 95% confidence intervals (95% CI) for specified groups. Spearman correlation analysis was used to examine the relationships between HPV infection and different age groups, high-risk (hr-HPV) subtypes, single and multiple hr-HPV subtype infections, and HPV persistence. Multivariate ordered logistic regression was used to analyse the characteristics of hr-HPV infection among populations with TCT-positive and pathological diagnosis-positive results. Differences were considered statistically significant when the *p*-value was less than 0.05.

## 3 Results

### 3.1 Prevalence of HPV infection

Out of 17,400 HPV screening tests conducted, 12.39% (2,155/17,400) of the samples tested positive for one or more HPV genotypes.

### 3.2 Age-specific distribution of HPV prevalence

**Figure 1:**
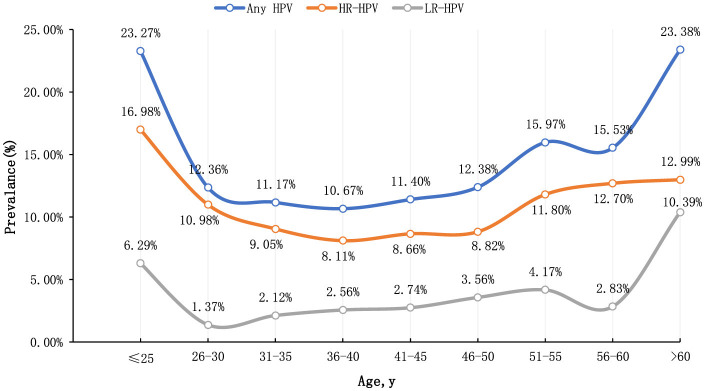
The prevalence of HPV among women in different age groups.

This study categorized participants by age, revealing that the age-specific prevalence of HPV exhibited a bimodal distribution. The infection rate was 23.27% (95% CI: 16.63%−29.91%) in individuals younger than 25 years and 23.38% (95% CI: 13.71%−33.05%) in those older than 60 years. Conversely, the lowest HPV infection rate was observed in women aged 36–40 years, at 10.67% (95% CI: 9.65%−11.68%). Additionally, the rate of high-risk human papillomavirus (hr-HPV) infection was 16.98% (95% CI: 11.08%−22.88%) in individuals under 25 years and 12.99% (95% CI: 5.31%−20.67%) in individuals over 60 years. Binary logistic regression analysis revealed significant differences in HPV infection rates across different age groups (*P* < 0.001), indicating statistical significance (Figure 1).

### 3.3 Distribution of hr-HPV genotypes in the general population

**Figure 2:**
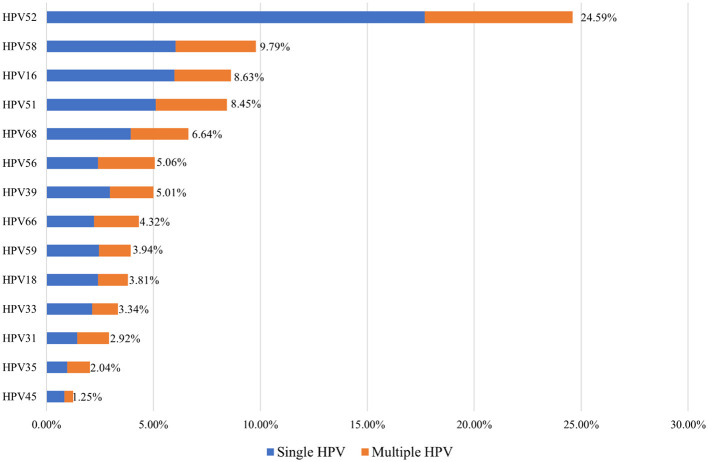
Comparison of single and multiple HPV infection rates.

Among the HPV-positive women in Shenzhen's Dapeng New District, the five most common hr-HPV genotypes were HPV52, HPV58, HPV16, HPV51, and HPV68 (24.59%, 9.79%, 8.63%, 8.45%, and 6.64%, respectively). Single hr-HPV genotype infections accounted for 78.75% (1,697/2,155) of the positive cases, while multiple hr-HPV genotype infections accounted for 21.25% (109/2,155) of the cases (Figure 2).

### 3.4 hr-HPV infection in TCT-positive population

**Figure 3:**
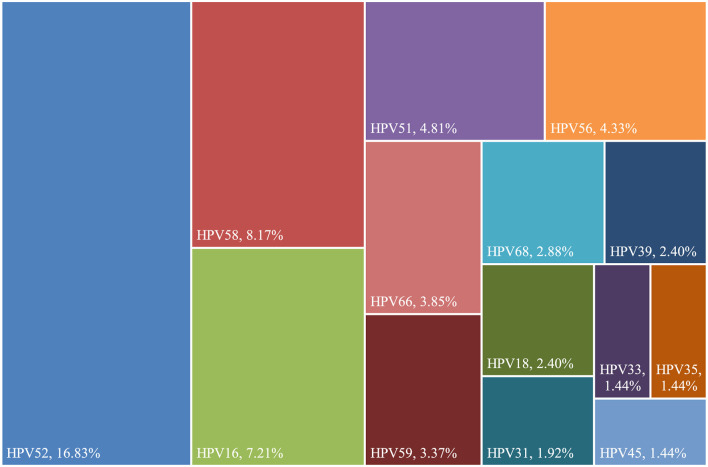
Distribution of high-risk HPV genotypes in 131 TCT-positive cases in Dapeng New District from 2020 to 2023.

Between January 2020 and December 2023, 5,044 TCT tests were performed in Dapeng New District, with 209 cases (4.14%) testing positive. The TCT-positive cases included ASC-US (2.85%), ASC-H (0.34%), LSIL (0.79%), HSIL (0.14%), and AGC- NOS (0.02%). The hr-HPV infection rate in TCT-positive cases was 53.59%, with the most common hr-HPV genotypes being HPV52, HPV58, HPV16, HPV51, and HPV56 (16.83%, 8.17%, 7.21%, 4.81%, 2.88%, and 4.33%) (Figure 3).

Among 209 patients with abnormal TCT results, 112 were positive for high-risk human papillomavirus (hr-HPV), with an infection rate of 53.59%. Among these, 30 cases (14.42%) had multiple infections. The hr-HPV positivity rate was 43.05% in patients with ASC-US. The most common hr-HPV genotypes detected were HPV52 (35.48%), HPV58 (14.51%), HPV16 (9.67%), HPV59 (9.67%), HPV51 (8.06%), HPV56 (8.06%), HPV66 (8.06%), HPV68 (4.83%), HPV39 (4.83%), HPV35 (4.83%), HPV18 (1.67%), HPV33 (1.67%, HPV31 (1.67%), and HPV45 (1.67%).

In patients with ASC-H, the hr-HPV positivity rate was 58.82%. The most common hr-HPV genotypes detected were HPV58 (40%), HPV52 (30%), HPV18 (20%), HPV16 (10%), HPV59 (10%), HPV33 (10%), and HPV31 (10%).

In patients with LSIL, the hr-HPV positivity rate was 80%. The most common hr-HPV genotypes detected were HPV52 (31.25%), HPV51 (12.5%), HPV58 (9.37%), HPV68 (9.37%), HPV56 (9.37%), HPV66 (9.37%), HPV16 (6.25%), HPV39 (6.25%), HPV18 (6.25%), HPV31 (6.25%), HPV33 (3.12%), and HPV45 (3.12%).

In patients with HSIL, the hr-HPV positivity rate was 100%. The most common hr-HPV genotypes detected were HPV16 (85.71%), HPV58 (14.28%), HPV51 (14.28%), and HPV56 (14.28%). No cases of AGC were identified (Supplementary Figure S1).

In patients with AGC- NOS, the hr-HPV positivity rate was 100%. The hr-HPV genotype detected were HPV45 (100%).

### 3.5 HPV infection in cervical cancer and precancerous lesions

**Figure 4:**
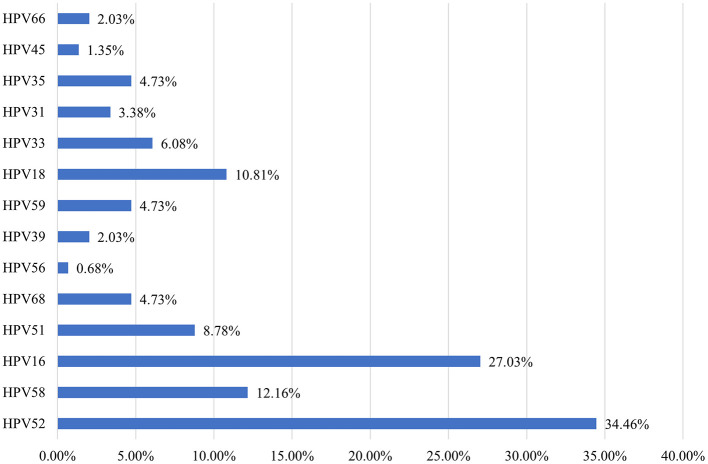
Distribution of high-risk HPV genotypes in 148 pathology-positive cases in Dapeng New District from 2020 to 2023.

A total of 291 participants underwent colposcopy and cervical histopathology, with 148 cases (49.14%) testing positive on histopathology. Among these, CIN I, CIN II/III, and CC were found in 29.90%, 20.96%, and 1.72% of cases, respectively. The hr-HPV infection rate in histopathology-positive cases was 95.95%, with the five most common hr-HPV genotypes being HPV52, HPV16, HPV58, HPV18, and HPV51 (34.46%, 27.03%, 12.16%, 10.81%, and 8.78%, respectively) (Figure 4).

Among patients with abnormal histopathological findings, the hr-HPV positivity rate was 95.95%, with 92 cases (31.94%) presenting multiple infections. In patients with LSIL on histopathology, the hr-HPV positivity rate was 94.25%, and the most common genotypes were HPV52 (37.8%), HPV16 (17.07%), HPV18 (15.85%), HPV51 (12.19%), HPV58 (9.75%), HPV68 (8.53%), HPV59 (6.09%), HPV31 (4.87%), HPV35 (4.87%), HPV33 (4.87%), HPV39 (2.43%), HPV66 (2.43%), HPV56 (1.21%), and HPV45 (1.21%).

In patients with HSIL on histopathology, the hr-HPV positivity rate was 98.21%, and the most common genotypes were HPV16 (43.63%), HPV52 (36.36%), HPV58 (16.36%), HPV33 (9.09%), HPV51 (5.45%), HPV35 (5.45%), HPV59 (3.63%), HPV18 (3.63%), HPV39 (1.81%), HPV66 (1.81%), and HPV31 (1.81%).

In patients with cervical cancer (CC) on histopathology, the hr-HPV positivity rate was 100%, with the most common genotypes being HPV16 (40%), HPV58 (20%), HPV18 (20%), and HPV45 (20%). No cases of AGC were identified (Supplementary Figure S2).

### 3.6 Impact of large-scale HPV vaccination on HPV infection trends

**Figure 5:**
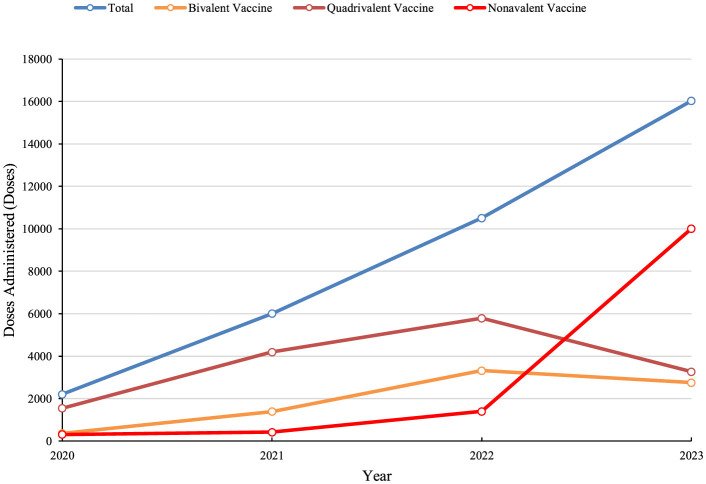
Annual trends in HPV vaccine doses administered in Dapeng New District from 2020 to 2023.

Between 2021 and 2023, the number of HPV vaccine doses administered to women of all ages in Dapeng New District steadily increased, reaching a totaling 34,748 doses. As the benefits of HPV vaccination became more widely recognized, bivalent and quadrivalent HPV vaccines were primarily used from 2020 to 2022. However, the adoption of the non-avalent HPV vaccine gained momentum after 2022, leading to a decline in the uptake of bivalent and quadrivalent vaccines (Figure 5).

**Figure 6:**
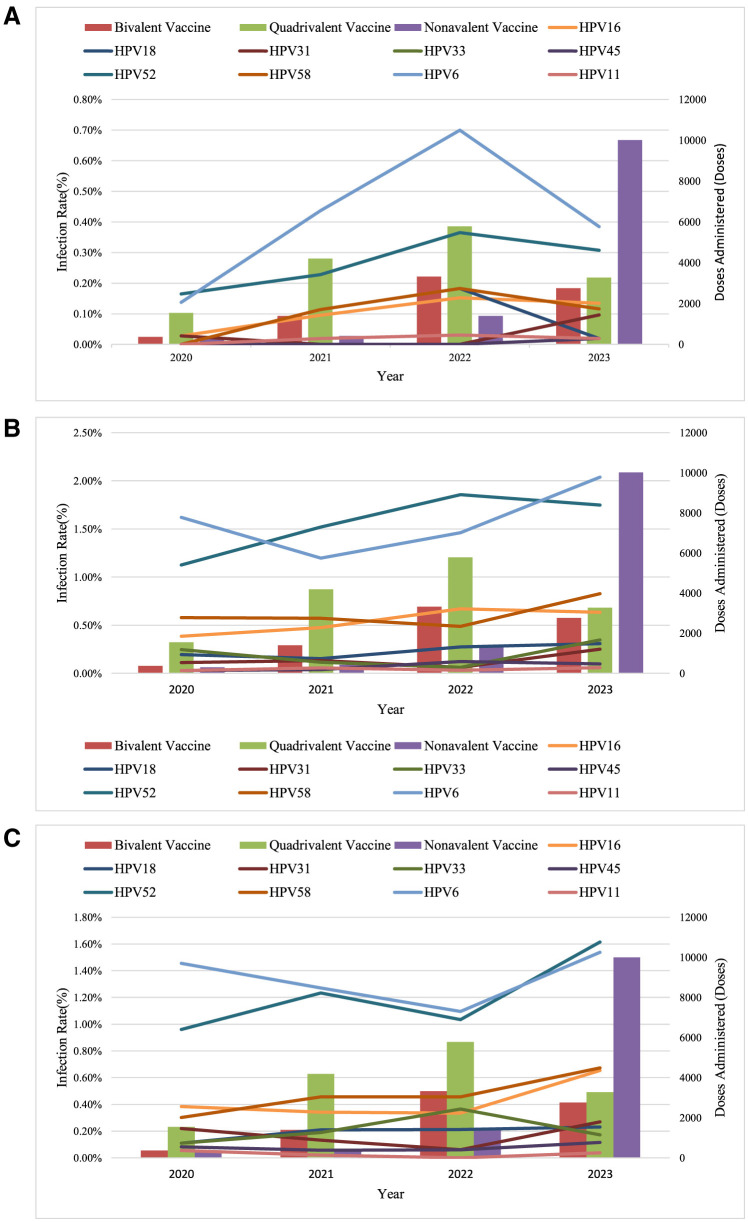
Trends in HPV infection among women in Dapeng New District (2020-2023). **(A)** Women under 30; **(B)** Women aged 31–45; **(C)** Women aged 46 and above.

To assess the protective effect of large-scale HPV vaccination on specific populations in Dapeng New District, considering that the early vaccine recipients were predominantly young women aged 9–24 years (Gynecological Oncology Society of Chinese Medical Association, 2021), we analyzed 17,400 HPV screening tests from 2020 to 2023 by dividing the population into three age groups: Group C1 (≤30 years), Group C2 (31–45 years), and Group C3 (>45 years). The annual trends in HPV 16, 18, 31, 33, 45, 52, 58, 6, and 11 infection rates were calculated for each group. From 2020 to 2022, an increase in infection rates for these HPV types was observed across all three groups. However, after 2022, a significant decline in HPV 16, 18, 31, 33, 45, 52, 58, and 11 infection rates was noted in Group C1, while in Group C2, only HPV 16, 18, 45, and 52 infection rates decreased, with HPV 31, 33, and 58 rates increasing. In Group C3, HPV 33 infection rates decreased, whereas infection rates for other HPV types increased (Figure 6).

## 4 Discussion

This study analyzed the results of 17,400 HPV screenings conducted among women in the Dapeng New District of Shenzhen from 2020 to 2023, revealing an HPV infection rate of 12.39%, which is lower than the overall HPV infection rate in China (15.0%) (Bao et al., 2021). Compared with other districts in Shenzhen, the overall HPV infection rates were reported to be 11.38% in Longgang District (Li et al., 2022), 12.62% in Luohu District (He et al., 2021), and 10.6% in Futian District (Ding et al., 2016). These findings indicate that the HPV infection rate in Dapeng New District is largely consistent with the overall HPV infection rate in Shenzhen.

The “Global HPV and Related Diseases” report indicates that HPV infection is closely related to age, with two infection peaks occurring at ages 17–24 and 40–44 (Zhao et al., 2012). In this study, the HPV infection rate among women also exhibited a bimodal distribution, with peaks observed at ages 20–25 and over 60. Given the higher HPV infection rate among women under 25, which is mostly transient with a low incidence of cervical cancer (Fontham et al., 2020), it is suggested that cervical cancer screening for women over 60 should be strengthened in this region.

The most common high-risk HPV (hr-HPV) genotypes detected in women from Dapeng New District were HPV52, 58, 16, 51, and 68 (24.59%, 9.79%, 8.63%, 8.45%, and 6.64%, respectively). The “Global HPV and Related Diseases” report indicates that the most common hr-HPV genotypes among women aged 20 and above in China are HPV52, 58, 53, 16, and 51 (Bao et al., 2021). This shows some differences in the most prevalent hr-HPV genotypes between women in Dapeng New District and the general population in China.

In this study, the hr-HPV infection rate was 53.37% among TCT-positive women and 95.95% among those with positive cervical histopathology. In China, the hr-HPV infection rates among women with normal cervical cytology, LSIL, HSIL, and invasive cervical cancer are reported to be 15.6%, 69.8%, 86.0%, and 88.7%, respectively (Ma et al., 2018). The significantly higher hr-HPV infection rate among women with positive cervical histopathology in this study may be attributed to the primary screening method used in this cohort, which was HPV genotyping triage (82.53%).

The five most common hr-HPV genotypes among TCT-positive women were HPV52, 58, 16, 51, and 68 (24.59%, 9.79%, 8.63%, 8.45%, and 6.64%, respectively). Among women with positive cervical histopathology, the five most common hr-HPV genotypes were HPV52, 16, 58, 18, and 51 (34.46%, 27.03%, 12.16%, 10.81%, and 8.78%, respectively). These findings provide scientific evidence for selecting the appropriate HPV vaccine types for women in this region.

The relationship between abnormal TCT results and hr-HPV infection rates shows that the hr-HPV positivity rate is 43.05% in ASC-US, 58.82% in ASC-H, 80% in LSIL, and 100% in HSIL. These data indicate that individuals with more severe cytological changes have higher hr-HPV infection rates.

The American ATHENA clinical trial reported an hr-HPV positivity rate of 32.6% in ASC-US patients (Stoler et al., 2012). In contrast, two large-scale clinical studies from hospitals in China showed hr-HPV positivity rates of 61.8% and 66.9% in ASC-US females, respectively (Jiang et al., 2022; Tao et al., 2022). Additionally, a study in China reported hr-HPV infection rates of 15.6% in women with normal cervical cytology, 69.8% in LSIL, 86.0% in HSIL, and 88.7% in invasive cervical cancer (Chen et al., 2009). A nationwide multicenter hospital-based study revealed that the hr-HPV infection rates in cervical squamous cell carcinoma and cervical adenocarcinoma were 97.6% and 74.5%, respectively (Chen et al., 2016).

These findings highlight significant regional differences in hr-HPV positivity rates among ASC-US patients. In our study, the hr-HPV positivity rates in LSIL and HSIL patients were notably higher than those reported elsewhere, which may be related to the small sample size of patients in our cohort.

Among patients with abnormal histopathological findings, the hr-HPV positivity rate was 95.95%, with 92 cases (31.94%) presenting multiple infections. The hr-HPV positivity rates were 94.25% in patients with LSIL, 98.21% in those with HSIL, and 100% in those with CC. The extremely high hr-HPV infection rates across all categories of histopathological abnormalities in this sample, especially among CC patients, may be influenced by the small sample size, affecting the objectivity of the statistical results.

The predominant hr-HPV genotypes in patients with LSIL were HPV52, 16, 18, 51, and 58, accounting for 71.67%. In patients with HSIL, the main hr-HPV genotypes were HPV16, 52, 58, 33, and 51, accounting for 85.89%. These findings show slight differences from other domestic studies. For instance, a study based on 17,311 individuals in China showed that in cervical intraepithelial neoplasia (CIN), the common genotypes for CIN1 were HPV16, 58, 33, 52, and 51, while for CIN2 and CIN3+, the common genotypes were HPV16, 58, 52, 33, and 31 (Dun et al., 2024).

In patients with CC, the main hr-HPV genotypes were HPV16 (40%), HPV58 (20%), HPV18 (20%), and HPV45 (20%), which are consistent with findings from other studies (Chen et al., 2009).

Regarding the impact of large-scale HPV vaccination on the trends in hr-HPV infection, this study observed a significant decline in the infection rates of HPV16, 18, 52, 58, 51, 31, 33, 45, 6, and 11 among women under 30 years of age in Dapeng New District as the overall HPV vaccination coverage increased. The infection rates of these HPV types also declined among women aged 31–45, while an increase was observed in women over 45 years of age, likely because the early vaccine recipients were predominantly young individuals aged 9–24 (Gynecological Oncology Society of Chinese Medical Association, 2021). The World Health Organization recommends that the optimal age for HPV vaccination is 9–14 years (World Health Organization = Organisation mondiale de la, 2022), and the Chinese consensus also prioritizes HPV vaccination for women aged 9–26, while recommending vaccination for women aged 27–45, which is applicable to this region as well. Since HPV vaccines do not cover all high-risk HPV genotypes and cannot prevent all HPV infections, and because vaccinated individuals who are sexually active may have already been infected with HPV before vaccination, HPV vaccination cannot interrupt the progression of an existing HPV infection. Therefore, regular screening should continue even after HPV vaccination (Gynecological Oncology Society of Chinese Medical Association, 2021).

With the increase in overall HPV vaccination coverage, a total of 34,748 doses of HPV vaccine were administered in Dapeng New District from 2020 to 2023, with 74.14% of these doses administered to women aged 9–35 years. After 2022, the infection rates of HPV types 16, 18, 52, 58, 51, 31, 33, 45, 6, and 11 significantly decreased among women under 30 years old, while the infection rates of HPV types 16, 18, 45, and 52 decreased among women aged 31–45 years. Conversely, the infection rates of HPV types 31, 33, and 58 increased in this age group. The reasons for these phenomena may include the following two possibilities: (1) limited data leading to statistical bias; (2) immune escape. Future research should include more data for statistical analysis to confirm these findings.

Among women over 45 years old, the infection rates of all hr-HPV subtypes increased, likely due to the lack of HPV vaccination coverage in this age group. Based on these findings, it is recommended that cervical cancer screening programs in this region prioritize women over 45 years old. Additionally, increasing the HPV vaccination rate among women is an effective measure, in line with health economics, to reduce the hr-HPV infection rate and cervical cancer incidence in this area.

This study had several limitations. First, the sample size is limited, particularly among individuals under 25 years old and over 60 years old, and this may compromise the accuracy of HPV infection rate estimates in these two critical age groups, thereby affecting the generalizability and extrapolation of the results. Additionally, reliance on historical medical records may introduce data gaps or incomplete documentation, potentially influencing the precise evaluation of HPV infection rates and associated factors. Moreover, this cross-sectional study lacks long-term follow-up information, precluding an accurate assessment of the natural history, progression, and dynamic relationship between HPV infection and cervical lesions. Future research should aim to increase sample size, optimize sample distribution, particularly among key populations, and incorporate prospective designs with long-term follow-up to validate our findings and address the current limitations. As 15,407 cases (82.53%) in this sample were triaged by HPV genotyping, with those positive for HPV16 and 18 directly referred to colposcopy without undergoing TCT testing, only one case of AGC was identified. This limited the study of HPV genotypes related to AGC in cytology.

## 5 Conclusion

In summary, the overall HPV infection rate in Dapeng New District, Shenzhen, is 12.39%. The HPV infection rate exhibits a bimodal distribution, with peaks in individuals under 25 years old (23.27%) and over 60 years old (23.38%). The five most common high-risk HPV (hr-HPV) types are HPV52 (24.59%), HPV58 (9.79%), HPV16 (8.63%), HPV51 (8.45%), and HPV68 (6.64%). For individuals with positive pathological findings, the most common hr-HPV types are HPV52, HPV16, HPV58, HPV18, and HPV51.

Based on the analysis of the correlation between the increase in HPV vaccination doses from 2020 to 2023 and changes in HPV infection rates across different age groups, it is recommended that cervical cancer screening programs in this region prioritize women over 45 years old. Additionally, increasing the HPV vaccination rate among women is an effective measure that is also in line with health economics to reduce the hr-HPV infection rate and cervical cancer incidence in this area.

## Data Availability

The raw data supporting the conclusions of this article will be made available by the authors, without undue reservation.
