# Supplementary material for: Cohort analysis of high-risk HPV infection in adult women in Dapeng New District, Shenzhen, Guangdong Province, China
Source: Front Microbiol. 2025 Mar 12;16:1539209. doi: 10.3389/fmicb.2025.1539209 (PMC11937043; doi:10.3389/fmicb.2025.1539209)
Supplement: Supplementary file 1 [file Data_Sheet_1.pdf]

Supplementary Material

Cohort Analysis of High-Risk HPV Infection in Adult Women in Dapeng New District, Shenzhen, Guangdong Province, China

Weifeng Wei[1]†, Mi Zhang[2]†, Yiyuan Lin[3], Ziyin Li[4], Wenbo Luo[5], Jing Zhuang[1], Weining Zhao[3], Zhixue Xu[3], Gaomin Li[3], Wenjing Zeng[1], Yan Tan [1*], Zhiying Yu [6*], Guanglei Li[3*]

[1]Shenzhen Dapeng New District Maternity and Child Health Hospital, Shenzhen, China

[2]Guangxi University of Chinese Medicine, Nanning, China

[3]Shenzhen Technology University, Shenzhen, China

[4]Shenzhen University, Shenzhen, China.

[5]Dapeng Center For Disease Control And Prevention, China

[6]Shenzhen Key Laboratory of Reproductive Immunology for Peri-implantation, Shenzhen Zhongshan Institute for Reproductive Medicine and Genetics, Shenzhen Zhongshan Obstetrics & Gynecology Hospital, Shenzhen, China

*** Correspondence:**
Yan Tan [1*]
504443109@qq.com
Zhiying Yu [6*]
lizheyzy@163.com
Guanglei Li[3*]
liguanglei@sztu.edu.cn

Keywords: Shenzhen Dapeng New District, Infection Rate, Human Papillomavirus (HPV) , Cervical Cancer Screening, Human Papillomavirus Vaccine Vaccination.

Supplementary Table S1: Impact of Redundant HPV Screening Records on Genotype-Specific Infection Rates

HPV Subtype	Total Cases	Original Rate (%)	Redundant Cases	Adjusted Rate (%)	Rate Difference (%)
HPV52	530	3.04%	30	2.87%	-0.17%
HPV58	211	1.21%	13	1.13%	-0.08%
HPV16	186	1.06%	9	1.01%	-0.05%
HPV51	182	1.04%	8	1.00%	-0.04%
HPV68	143	0.82%	6	0.78%	-0.04%
HPV56	109	0.62%	9	0.57%	-0.05%
HPV39	108	0.62%	8	0.57%	-0.05%
HPV66	93	0.53%	9	0.48%	-0.05%
HPV59	85	0.48%	5	0.45%	-0.03%
HPV18	82	0.47%	6	0.43%	-0.04%
HPV33	72	0.41%	8	0.36%	-0.05%
HPV31	63	0.36%	6	0.32%	-0.04%
HPV35	44	0.25%	2	0.24%	-0.01%
HPV45	27	0.15%	2	0.14%	-0.01%
Total	1935	11.12%	121	10.42%	-0.70%

Supplementary Figure S1. Distribution of High-Risk HPV Genotypes in TCT-Positive Individuals with Different Cytological Diagnoses: (A) Distribution of High-Risk HPV Genotypes in ASC-US Cases; (B) Distribution of High-Risk HPV Genotypes in ASC-H Cases; (C) Distribution of High-Risk HPV Genotypes in LSIL Cases; (D) Distribution of High-Risk HPV Genotypes in HSIL Cases

(A)

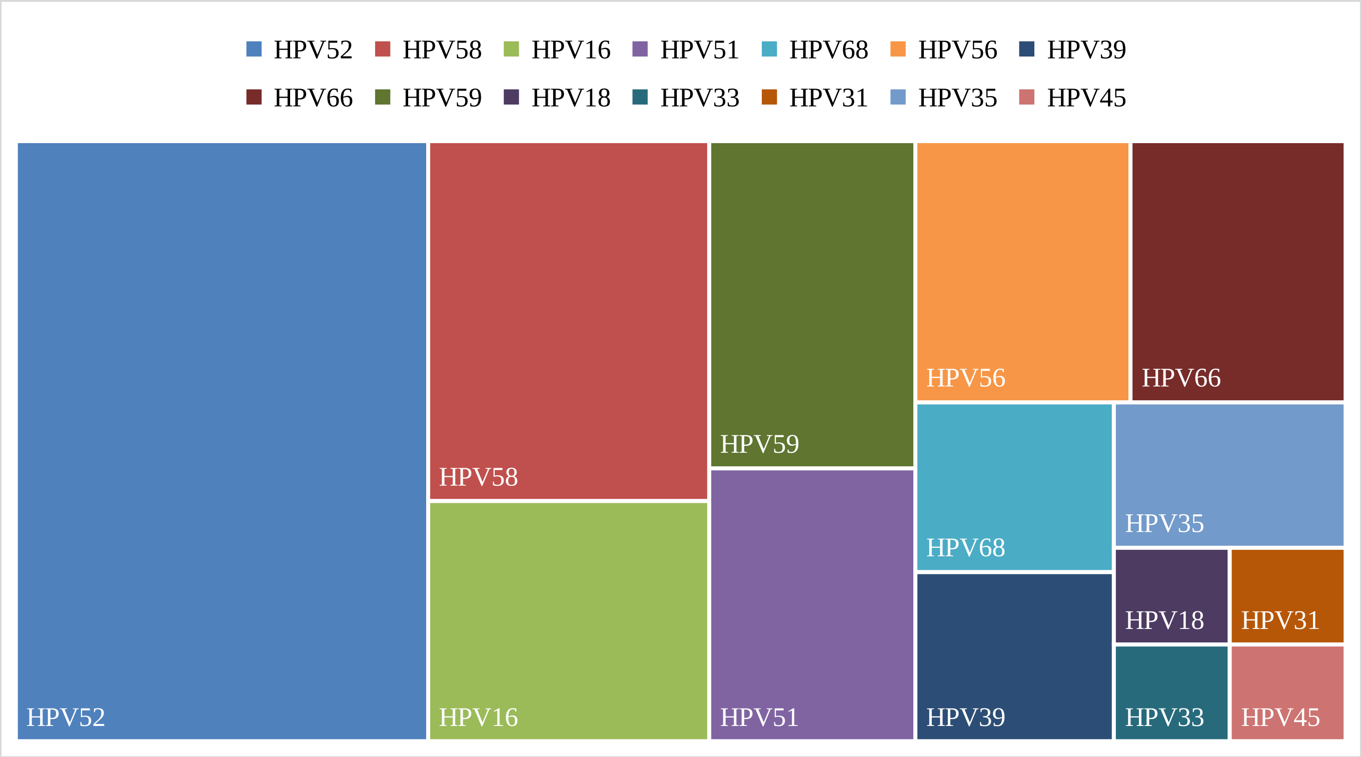
HPV52
HPV58
HPV16
HPV51
HPV68
HPV56
HPV39
HPV66
HPV59
HPV18
HPV33
HPV31
HPV35
HPV45
HPV52
HPV58
HPV16
HPV59
HPV51
HPV56
HPV66
HPV68
HPV35
HPV39
HPV18
HPV31
HPV33
HPV45

(B)

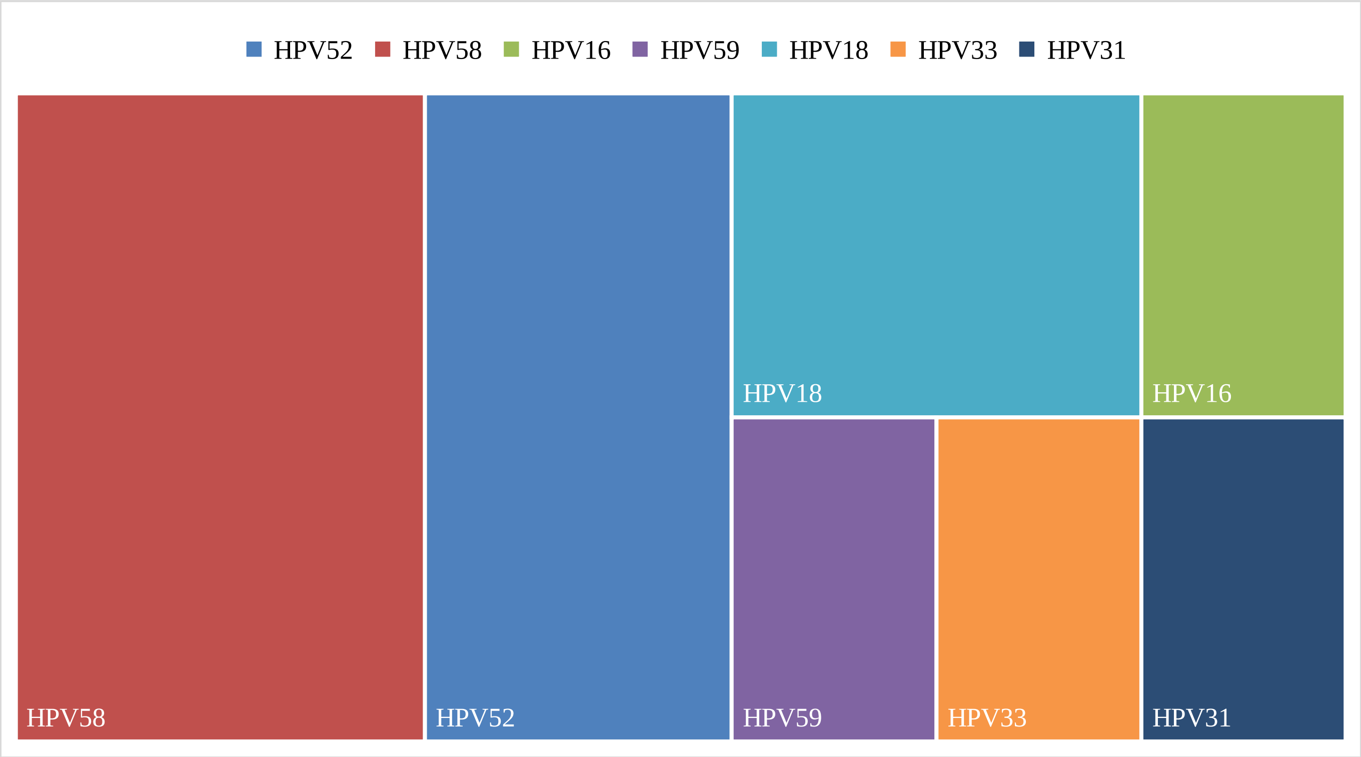
HPV52
HPV58
HPV16
HPV59
HPV18
HPV33
HPV31
HPV58
HPV52
HPV18
HPV16
HPV59
HPV33
HPV31

(C)

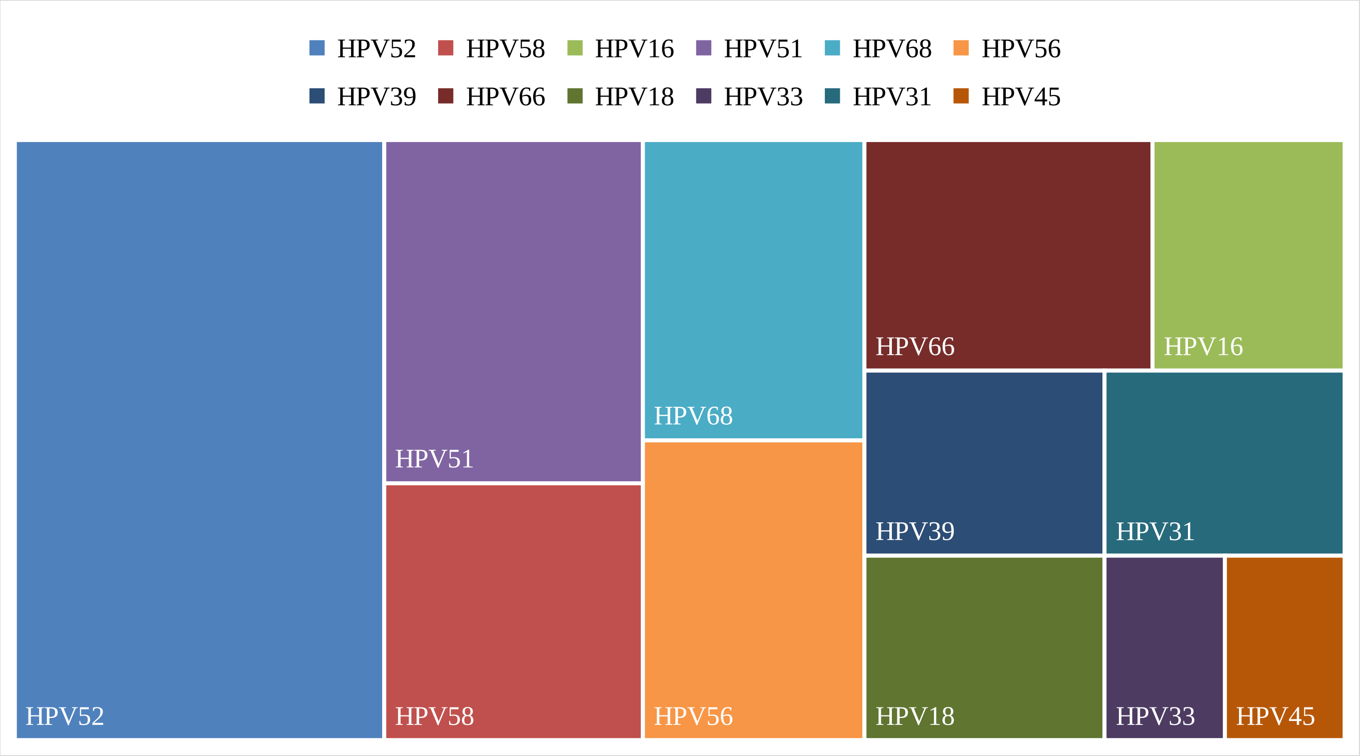
HPV52
HPV58
HPV16
HPV51
HPV68
HPV56
HPV39
HPV66
HPV18
HPV33
HPV31
HPV45
HPV52
HPV51
HPV58
HPV68
HPV56
HPV66
HPV16
HPV39
HPV31
HPV18
HPV33
HPV45

(D)

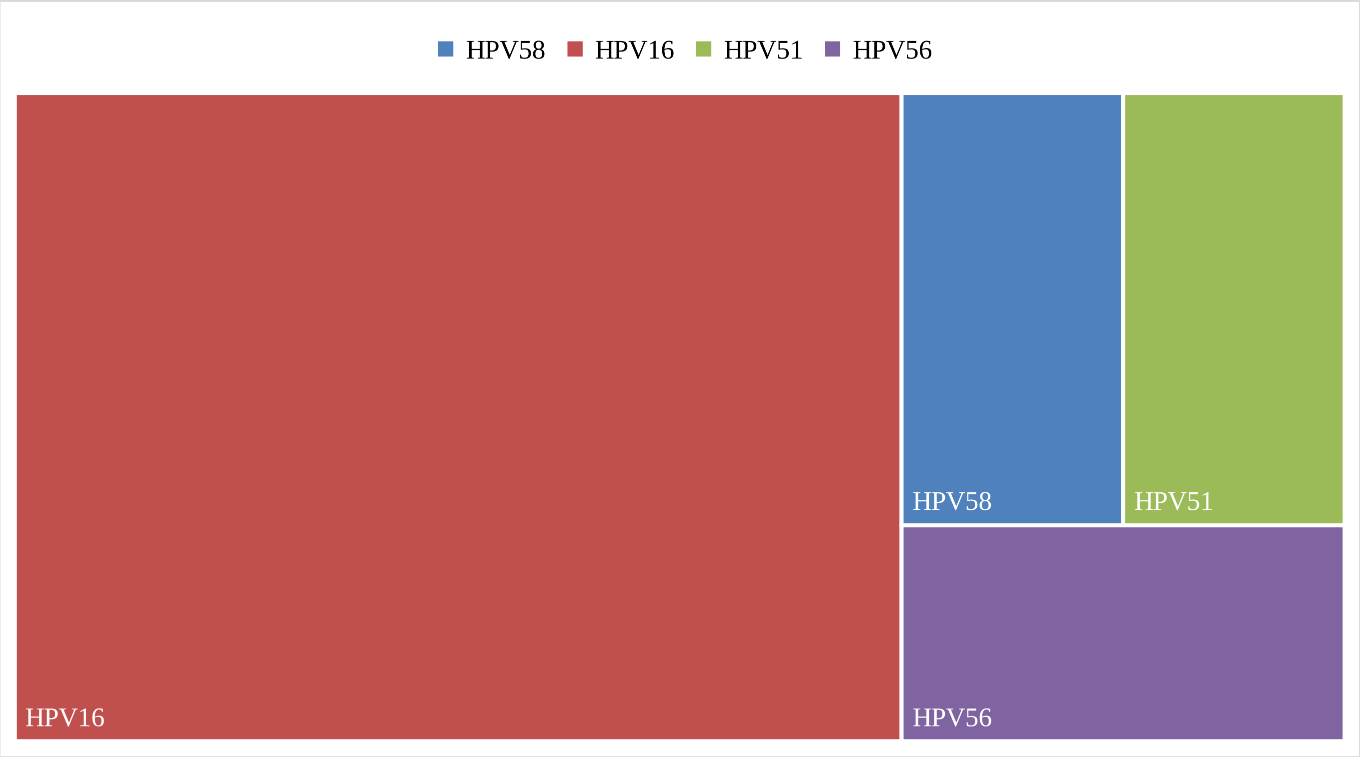
HPV58
HPV16
HPV51
HPV56
HPV16
HPV58
HPV51
HPV56

Supplementary Figure S2. Distribution of High-Risk HPV Genotypes in Histologically Positive Individuals: (A) Proportion of High-Risk HPV Genotypes in LSIL Cases; (B) Proportion of High-Risk HPV Genotypes in HSIL Cases; (C) Proportion of High-Risk HPV Genotypes in CC Cases

(A)

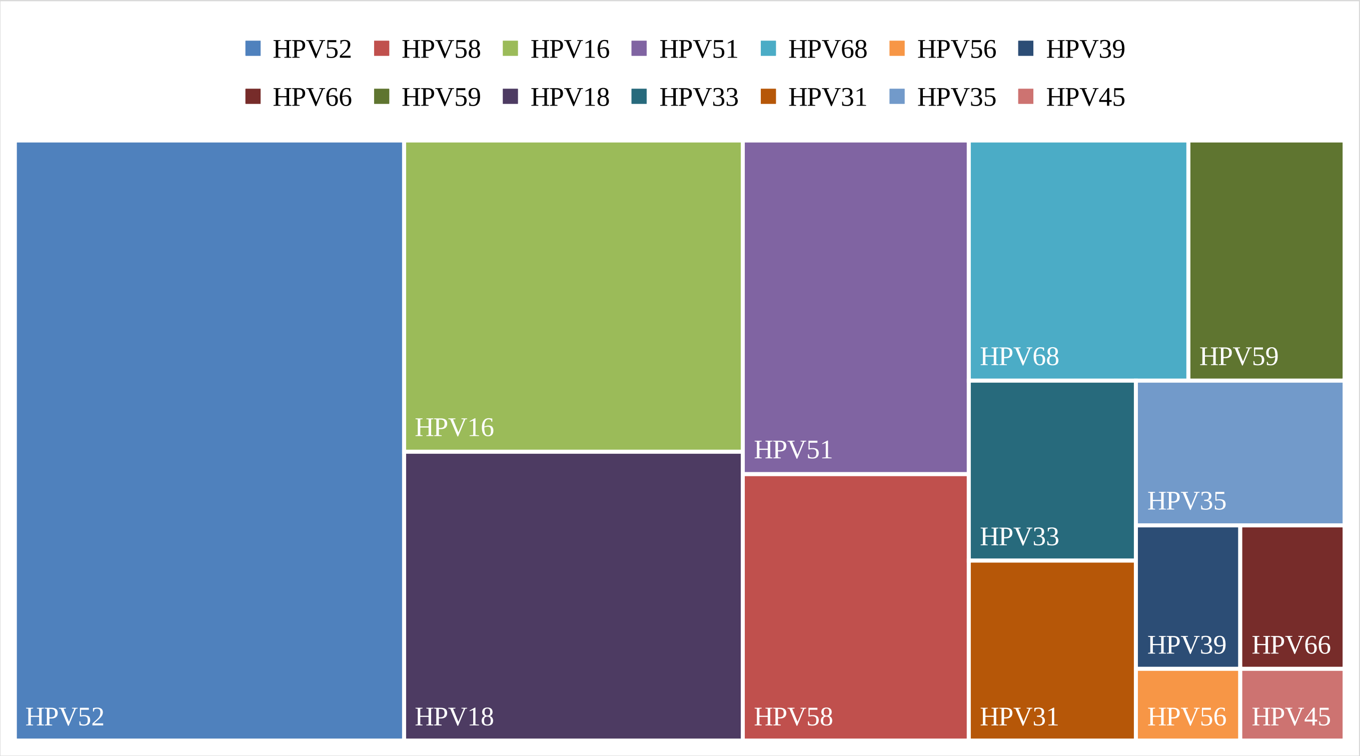

(B)

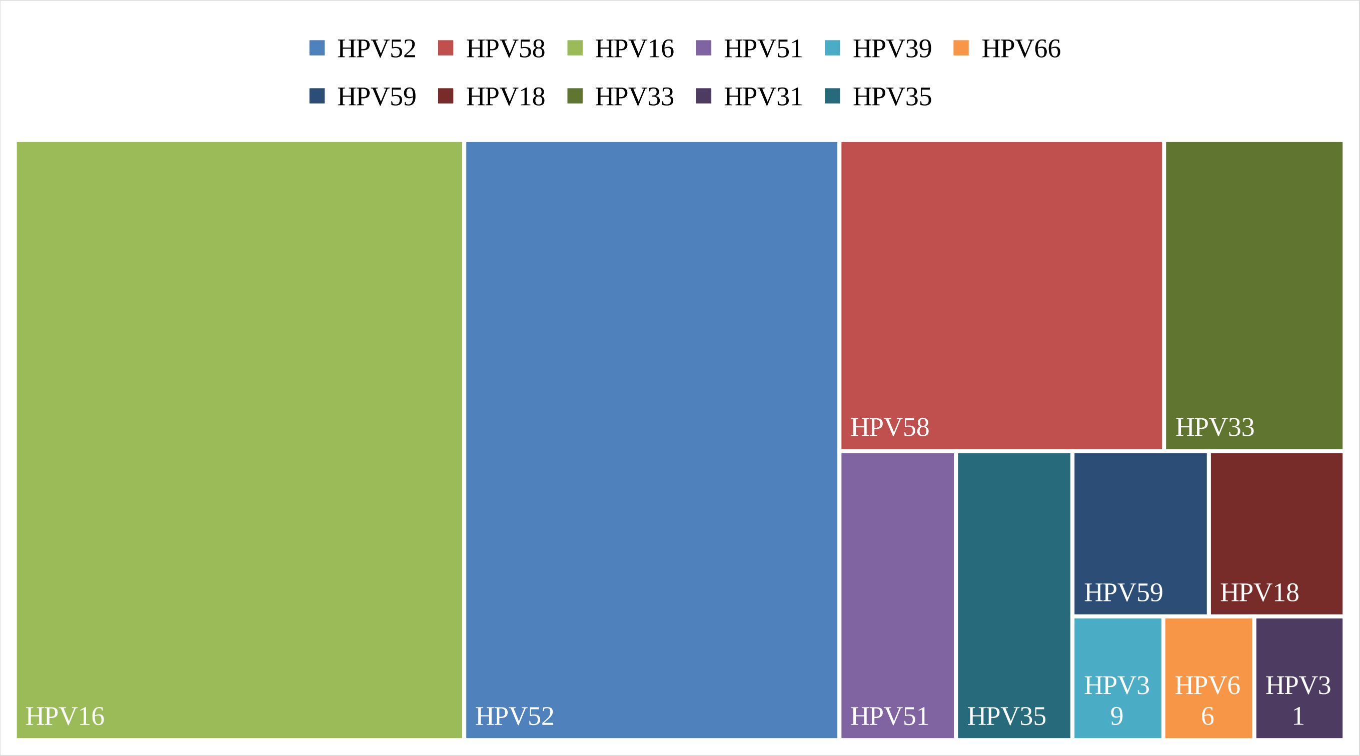
HPV52
HPV58
HPV16
HPV51
HPV39
HPV66
HPV59
HPV18
HPV33
HPV31
HPV35
HPV16
HPV52
HPV58
HPV33
HPV51
HPV35
HPV59
HPV18
HPV39
HPV66
HPV31

(C)

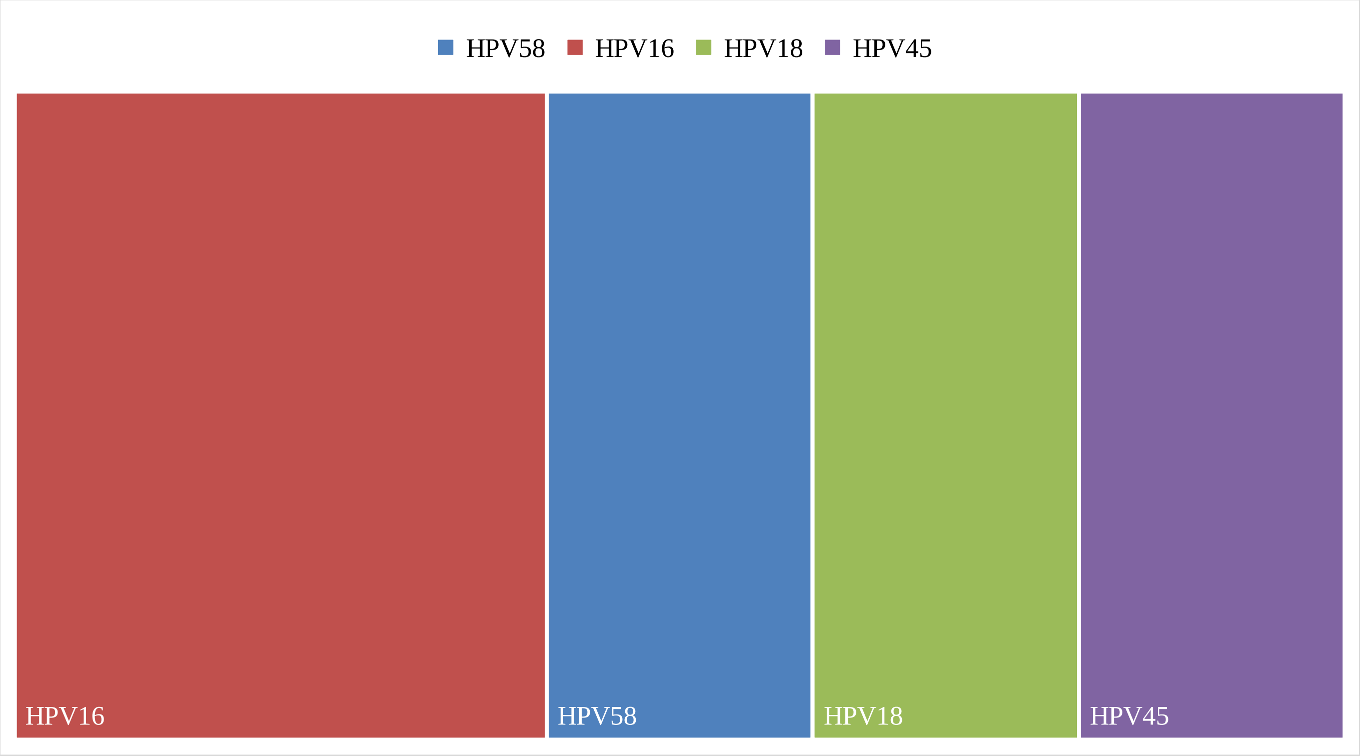
HPV58
HPV16
HPV18
HPV45
HPV16
HPV58
HPV18
HPV45
